# The Effect of the More Active MuMs in Stirling Trial on Body Composition and Psychological Well-Being among Postnatal Women

**DOI:** 10.1155/2016/4183648

**Published:** 2016-08-16

**Authors:** Alyssa S. Lee, Rhona J. McInnes, Adrienne R. Hughes, Wendy Guthrie, Ruth Jepson

**Affiliations:** ^1^School of Health Sciences & Sport, University of Stirling, Stirling FK9 4LA, UK; ^2^School of Nursing, Midwifery and Social Care, Edinburgh Napier University, Edinburgh EH11 4BN, UK; ^3^School of Psychological Sciences and Health, University of Strathclyde, Glasgow G1 1QE, UK; ^4^Scottish Collaboration for Public Health Research & Policy, University of Edinburgh, Edinburgh EH8 9DX, UK

## Abstract

*Introduction.* Physical activity is important for health and well-being; however, rates of postnatal physical activity can be low. This paper reports the secondary outcomes of a trial aimed at increasing physical activity among postnatal women.* Methods.* More Active MuMs in Stirling (MAMMiS) was a randomised controlled trial testing the effect of physical activity consultation and pram walking group intervention among inactive postnatal women. Data were collected on postnatal weight, body composition, general well-being, and fatigue. Participants were also interviewed regarding motivations and perceived benefits of participating in the trial.* Results.* There was no significant effect of the intervention on any weight/body composition outcome or on general well-being at three or six months of follow-up. There was a significant but inconsistent difference in fatigue between groups. Qualitative data highlighted a number of perceived benefits to weight, body composition, and particularly well-being (including improved fatigue) which were not borne out by objective data.* Discussion.* The MAMMiS study found no impact of the physical activity intervention on body composition and psychological well-being and indicates that further research is required to identify successful approaches to increase physical activity and improve health and well-being among postnatal women.

## 1. Introduction

Recent research suggests that physical inactivity in women over 30 years is the greatest preventable risk factor for cardiovascular disease [1]. Weight retention is a clinical problem among postnatal women and may be important in terms of lifetime obesity risk [2, 3]. Regular physical activity may contribute to short-term improved weight control (during and after pregnancy), longer-term overweight and obesity management, and diabetes treatment and prevention [4–6]. Also during the postnatal period psychological well-being has been shown to be enhanced by regular physical activity [7–9]. Recommendations for postnatal physical activity are now available via clinical guidelines published in five countries [10]. All promote the safety of physical activity and the beneficial effects of following generic guidelines for adults, with variable suitable periods suggested for gradual resumption (or uptake) of physical activity after birth.

Rates of postnatal physical activity participation vary considerably across studies but in general a low proportion of postnatal women report meeting physical activity guidelines [11–15]. In these studies there is considerable heterogeneity with regard to measurement approaches, the postnatal time period under study, and different definitions regarding what constitutes physical activity. Previous research suggests childbirth, pregnancy, and childrearing act as potentially negative influences on physical activity participation among women [16–18], partly due to increased caregiving responsibilities and societal and individual perceptions, particularly on how women prioritise their own needs in the context of their parental and/or working mother roles [19]. Qualitative evidence from mothers of young children lends some support to this [19, 20], as do surveys of postnatal women's self-reported PA barriers, which identify lack of time [21], lack of childcare, and low energy levels as the most frequently reported barriers [21–23]. Postnatal women reporting fewer barriers are more active [19], and self-reported self-efficacy (confidence) for overcoming barriers to PA (including setbacks to implementing PA plans) can predict positive changes to postnatal PA behaviour [22].

Our review and meta-analysis of postnatal interventions identified some evidence of a moderate positive effect on frequency of physical activity participation among postnatal women who received physical activity interventions [24]. Efficacious studies (in terms of physical activity outcomes) were generally those omitting dietary components and those utilising theoretically sound and evidence-based behavioural techniques such as goal-setting and self-monitoring. However, these studies did not report on other outcomes (e.g., weight management and indicators of postnatal well-being). Methodological flaws such as a poor sample size and lack of an objective measure of physical activity suggested further research was warranted. We designed, developed, and implemented the More Active MuMs in Stirling (MAMMiS) trial to address this underresearched area.

The primary aim of MAMMiS was to investigate the effect of an intervention comprising physical activity consultations and a 10-week pram walking programme on objectively measured physical activity in healthy but insufficiently active postnatal women. Main trial results are reported elsewhere [25]. This paper reports on secondary outcomes, with a particular focus on postnatal health and well-being indicators (specifically weight management, well-being, and fatigue). Although the MAMMiS trial was not designed as a postnatal weight management intervention (weight and body composition were secondary outcomes), this together with postnatal well-being outcomes is of importance to researchers, clinicians, and postnatal women themselves. The qualitative phase (aspects of which are reported here and explored in more detail in [26]) also considered intervention feasibility and acceptability from the perspective of the postnatal women participating in the trial.

## 2. Materials and Methods

MAMMiS study methods have been reported in detail elsewhere [27]. In this paper we present brief details of the central features of study design, participants recruited, and intervention and outcome measures of interest for this paper.

### 2.1. Design and Setting

MAMMiS was a randomised controlled trial conducted in one region within Central Scotland. The community has approximately 3–3500 births annually [28] and is reasonably diverse in terms of socioeconomic status and urban and rural classification, although ethnic minorities are underrepresented, which can be problematic for recruitment of a diverse sample [29].

### 2.2. Participants

Postnatal women who had given birth between 6 weeks and one year previously were included in the study. Inclusion criteria are given in full in Gilinsky et al. [27]. In brief, postnatal women (following postnatal check-up) who were insufficiently active (defined in relation to their self-reported physical activity stage of change [30], which assessed their current physical activity in relation to physical activity guidelines [31]) were included. Postnatal women were excluded if they were pregnant/planning a pregnancy or had medical contraindications to physical activity.

### 2.3. Intervention and Control Procedures

The intervention was delivered by a health psychologist with experience in motivational interviewing and delivery of behaviour change (plus walk leader training) and consisted of a face-to-face physical activity consultation (approximately 45 minutes in length) delivered at the start of a 10-week group pram walking programme with a second consultation (approximately 25 minutes in length) delivered at the end of the programme. The physical activity consultation approach was theoretically and evidence based (e.g., [32–34]) and was derived from models of behaviour change, in particular the Transtheoretical Model (TTM) [35]. The specific behaviour change techniques used in the physical activity consultations have been reported in detail elsewhere [27] and were chosen following a review of literature on determinants of postnatal physical activity. A workbook was used by participants to structure their activity plan (e.g., goal-setting, planning, and self-monitoring sheets). Participants also received a pedometer to monitor steps and were given information on the pram walk programme in their area. Participants could attend one session/week for 10 weeks and all women were encouraged to attend. Walks were conducted at a moderate-intensity (e.g., brisk pace) between 30 to 55 minutes per session. Four women who were unable to attend (e.g., because they had an infant and a toddler) received a 10-minute support phone call instead.

The control group received a leaflet “Active Living during and after Pregnancy,” an NHS Health Scotland publication with information on physical activity guidelines and advice on implementation.

### 2.4. Outcome Measures

The primary outcome measure for the MAMMiS study was change in physical activity (measured by accelerometry and questionnaire), which has been reported elsewhere [25, 27]. Secondary outcomes were weight, body mass, general well-being, and fatigue and these were assessed at baseline and three-month and six-month follow-up. Weight (kg) and body composition (BMI, % fat mass) were measured using the Tanita 300 MA portable bioelectrical impedance monitor in accordance with procedures specified in the technical manual [36]. Each test was conducted at the same time of day and participants were given instructions to improve the accuracy of the body composition measurements. Before each test participants were asked to avoid caffeine for four hours, eating and drinking for 4 hours, intense exercise for 12 hours, taking diuretics for 7 days, and alcohol for 48 hours; participants were also asked to empty their bladder within 30 minutes before the test. Height was measured in centimetres (to the nearest cm) using a stadiometer at baseline only. Participants were categorised as underweight, healthy weight, overweight, or obese according to their BMI (kg/m^2^). Psychological well-being was measured using the Adapted General Well-Being Index (AGWBI) [37]. This 22-item 5-point Likert response scale assesses well-being, self-control, anxiety and depression, vitality, and general health concerns in the past two weeks and has been validated within a GP practice in the UK [38]. Fatigue was measured using a visual analogue scale (VAS) response to one question. Visual analogue scales are a commonly used unidimensional method of assessing health status and are appropriate for measuring experience of short-term fatigue severity in general and clinical populations [39]. Participants were asked to place a mark on a 100 mm line to indicate the extent to which they had been “affected by fatigue in the past two weeks,” where no fatigue was equal to 0 and worst possible fatigue was equal to 100 on the VAS. All measurements were taken at participants' homes or at the university site, depending on participant preference.

### 2.5. Posttrial Interviews

For the qualitative phase participants from each trial group were sought and we aimed to recruit a representative sample of at least half of all MAMMiS participants. The qualitative phase consisted of one in-depth interview (30–90 minutes in length). These took place after completion of all outcome measures at participants' homes or another suitable venue. All interviews were conducted by a separate researcher not involved in the main trial (who also led the qualitative analysis). The rationale for this was to create an open atmosphere to explore trial experiences and assess acceptability of the intervention. A topic guide was developed to guide the interview; however participants were also encouraged to raise issues important to them. All interviews were tape-recorded and transcribed verbatim.

### 2.6. Analysis

*(i) Quantitative Data.* Mann-Whitney *U* tests were used to analyse differences between the intervention and control group for changes in weight and body composition from baseline to 3 months and from 3 to 6 months, as these outcomes were not normally distributed. Psychological well-being and fatigue were analysed using independent and paired-samples *t*-tests to investigate differences between the intervention and control group on changes in these outcome measures between baseline and three months (the intervention period) and between baseline and six months (follow-up period). All statistical analyses were discussed and agreed with an independent statistician.

*(ii) Qualitative Data.* Posttrial interviews with study participants were coded using NViVO qualitative analysis software to manage the dataset. Thematic analysis based on the approach described by Braun and Clarke [40] was used to analyse, iteratively code, and build up a final set of themes and subthemes. Two people coded a sample of interviews prior to compiling a final list and then quotes were extracted to exemplify themes. For the purpose of this paper the results will report on the following areas explored during interviews: reasons for participating in the trial (i.e., motivating factors) and belief in personal health and well-being benefits acquired from taking part in the trial (from a perceived increase in physical activity or other change, e.g., in secondary outcomes or motivations for being active).

## 3. Results

### 3.1. Participant Characteristics

Baseline characteristics for the sample are shown in Table 1. Postnatal women who enrolled in the MAMMiS study were on average 33 years of age with their youngest child averaging 24 weeks. Most participants were primiparous, married, degree-educated, and on maternity leave at baseline. Most study participants had given birth vaginally. Changes in weight and BMI following pregnancy were evident; average weight gain was around 4.5 kg from prepregnancy (self-reported) to enrolment in the study (measured weight). There appeared to be differences in baseline weight between the two study groups, with intervention participants being heavier and more likely to be overweight/obese (OW/OB). Control participants were more likely to be breastfeeding at baseline. None of the differences were statistically significant.

Although the group of women expressing interest in joining the study were representative of postnatal women in Scotland in respect to their age, deprivation, and urban/rural classification, women actually enrolling in the study were more likely to be from affluent Scottish Index of Multiple Deprivation (SIMD) areas [40] (data not shown) and were older (only 18% were under 30 years) compared with the total number of women who expressed an interest in joining the study.

Trial participants (*n* = 35) who completed posttrial interviews were representative of the main trial sample in terms of their number of children, postnatal stage at study onset, BMI classification at study onset, and whether they remained or dropped out of the study [26].

### 3.2. Study Flow

**Figure 1:**
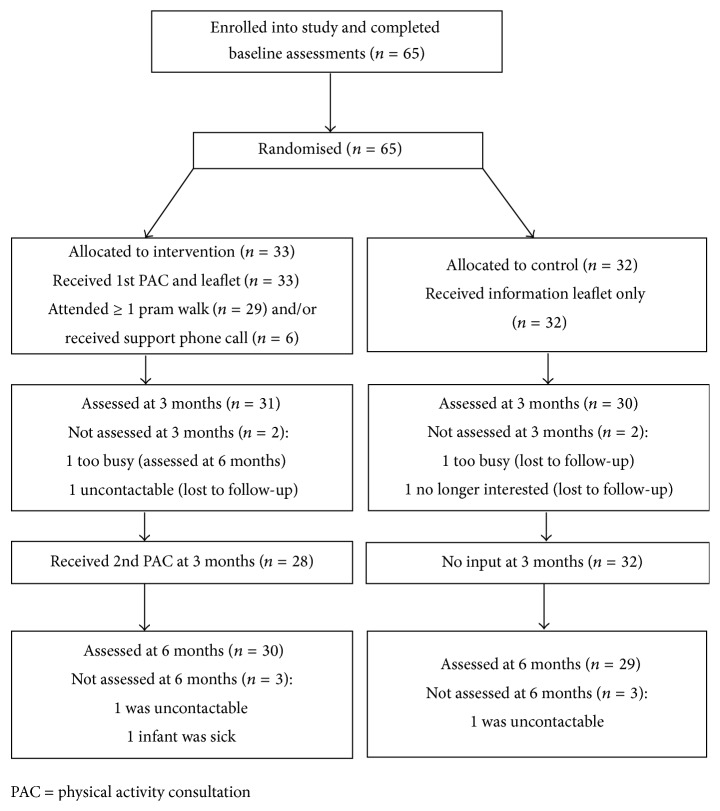
Flow of participants through the MAMMiS study.

All 65 participants completed baseline assessments and were randomised to the intervention or control group. All received the intended intervention or control condition and 92% (60/65) completed assessments at three months. Twenty-nine of the 33 intervention participants attended at least one pram walk, with the average number of walks attended being five (s.d. = 3.13) out of possible ten walks. At six-month follow-up 91% (59/65) of the sample completed assessments (see Figure 1). The number of participants not completing at least one assessment period (defined as withdrawals) was similar across the groups with no evidence that withdrawals differed from nonwithdrawals on baseline physical activity “stage of change,” weight status, SIMD, or number of children at home, although withdrawals were younger and had a younger baby (data not shown).

### 3.3. Effect of the Intervention on Postnatal Weight and Body Composition Outcomes

**Figure 2:**
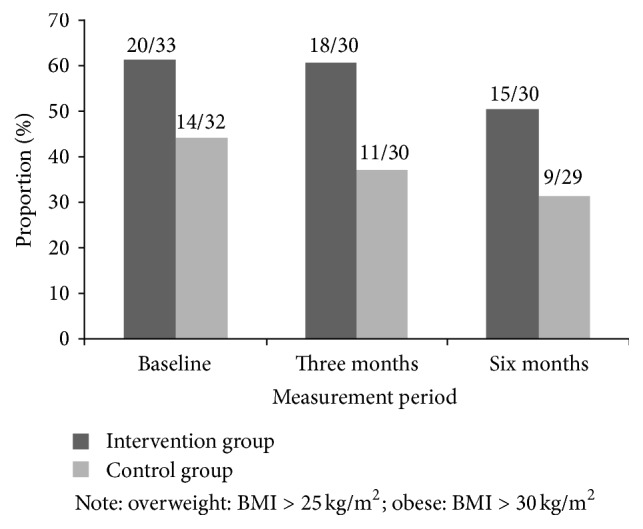
Proportion of overweight and obese participants at baseline and three and six months in response to a 10-week physical activity intervention.

Table 2 shows the median and interquartile range for weight, BMI, fat mass, and % fat mass at all measurement points during the study. There was no significant effect of the intervention on any weight/body composition outcome at three- or six-month follow-up (Table 2). Both groups showed a similar small decrease in weight/body composition outcomes from baseline to three months and from three to six months; however these time effects were associated with large confidence intervals and changes were not significant (Table 2). All outcomes remained higher in the intervention group compared with controls at all measurement points. Some participants did show a clinically significant change in BMI status over the six-month study period (Figure 2); that is, 25% (*n* = 5) of the 20 participants in the intervention group and 50% (*n* = 7) of the 14 participants in the control group who were overweight or obese at baseline went from obese to overweight or overweight to normal weight. Due to the small sample of overweight/obese participants this was not tested statistically.

### 3.4. Effect of the Intervention on Postnatal Psychological Well-Being and Fatigue Severity

Over the study period there was little evidence of an effect of the intervention on psychological well-being; that is, there were no significant between groups' differences from baseline to three- (*p* = 0.09; 95% CI −0.77, 10.95) and three- to six-month follow-up (*p* = 0.19; 95% CI −9.68, 1.97) (see Table 3).

Fatigue decreased in the intervention group from baseline to three months, while among control group participants fatigue increased from baseline to 3 months; this difference between the groups was significant (*p* < 0.01; 95% CI −36.49, −9.14). However, this pattern was reversed from three to six months, with fatigue increasing among intervention participants and decreasing among controls (*p* < 0.01; 95% CI 5.20, 34.86). Note: change measures were analysed using *t*-tests as these were normally distributed; however the median and IQ range scores are given in Table 4 as there was evidence of skew at each measurement point.

### 3.5. Posttrial Interviews

*Personal Reasons for Participating in the Trial and Wanting to Be Active.* A variety of reasons were given for joining the trial. Many mentioned weight management and the role that physical activity can play in relation to losing weight, “*I put loads of weight on, and I was really inactive all through my pregnancy, and I hated it, and I really wanted to, like start doing more, like exercise,*” and the possibility of increasing activity to compensate for eating habits, “*I like to keep my weight down and I love to eat loads of nice things…and I find that the more activity I do, you can have these treats more.*”

However, motivations for joining the trial were also related to the perceived general health benefits of pursing a more active lifestyle, including relaxation/mood improvement, “*the long-term reason is that I think it benefits your health. And I think it increases, in terms of, it improves your mood.*” Some postnatal women mentioned using activity to manage stresses of motherhood, “*I used to do yoga and stuff, that was very relaxing…I really need the time to myself,*” and/or to be a role model or better mother: “*it is quite important for these two to see from early – we try to go for family walks at the weekend…so they get used to it. My mum's not one for being active.*”

### 3.6. Belief in Benefits Gained by Becoming More Active as a Result of Joining the Trial

Regardless of group allocation in the trial, many participants perceived a benefit from being in the study and many felt they had become more active as a consequence of participating in the trial. When describing their personal benefits gained from increasing physical activity during the trial, one participant described feeling “*fitter and less fat.*” However, for many participants their perception of the value of physical activity appeared to relate to the importance of physical activity for addressing day-to-day challenges to their well-being, such as through increasing their energy levels/stamina, promoting good sleeping habits, and improving mood/releasing stress, “*more energy, without a doubt, sleep better, definitely helps mood I think. Kind of feel less tense – just overall well-being.*” Some participants reported unanticipated benefits related to being active since joining the trial; these also tended to focus on the benefits of activity to enable participants to cope with their life as a mother, “*I think the fresh air and getting out every day walking, we had a nice structure to our day. That helped me mentally, you know, just relax, not worry. It's my first baby and you spend a lot of time worrying, in general. Lots of worrying and I think that helped.*”

Unanticipated benefits were also reported by one mother reflecting on her return to work, “*The nature of my job is extremely stressful and so busy throughout the day, that I love the space of getting out on my own and having time – I find a lot of my best ideas come from that space and actually getting away from it all, just to be alone with your thoughts,*” although both work-life and mothering presented specific challenge in terms of making time for physical activity, “*I feel there's always something to be done and I find it hard, and it always will be there's never going to come a point when I find oh yes I've got a spare hour to go to the gym or something. I think women find it harder to cut off from what is needing to be done.*”

## 4. Discussion

The MAMMiS study found no impact of the physical activity intervention on secondary health outcomes for postnatal women. Changes in weight and body composition, along with general psychological well-being, were not significantly different between the postnatal women receiving a physical activity consultation and taking part in pram walking and those receiving an NHS leaflet. There was a significant positive impact on fatigue at three-month follow-up but this was not sustained at six months.

### 4.1. Lack of Significant Effects on Weight and Body Composition

The absence of a significant impact on weight/body composition-related secondary outcomes could be explained by the lack of a significant effect on objectively measured physical activity [25]. The small changes in weight/body composition in our study groups may indicate a natural return towards prepregnancy weight or may be due to the fact that over a third of women in both groups reported engaging in dietary control strategies to manage their weight. There is evidence that physical activity trials lacking a dietary component have little effect on postnatal weight outcomes [24] and among women in more general trials [41]. While weight management or concerns about body composition motivated some participants to take part in our trial [26] this may not be a good indicator of the sustained effort required to maintain a physically active lifestyle [42]. Participants in our trial also tended to be more affluent postnatal women who are less likely to experience long-term postnatal weight retention [43, 44] and more than half were of healthy prepregnancy weight suggesting less opportunity to demonstrate effect.

### 4.2. Lack of Significant Effects on Psychological Well-Being

MAMMiS was the first study to consider the impact of a physical activity consultation combined with group pram walking on general psychological well-being in healthy postnatal women. Although participants discussed concerns for their well-being as a motivator for taking part in the trial, psychological well-being remained stable over time in both groups with no evidence of an impact of the intervention. This may reflect the absence of change in physical activity or the relatively high psychological well-being at baseline. There is some evidence that group physical activity interventions, including pram walking, can improve well-being or postnatal depression scores in healthy postnatal women [45] and in women with postnatal depression [46, 47]. However, it is unclear whether these outcomes are attributable to changes in physical activity or to the addition of social support provided via group exercise, which is an ongoing issue in the postnatal physical activity literature [8]. There is some suggestion from our qualitative study that participating in the trial improved participants' perception of well-being despite a lack of increased objectively measured physical activity. A pilot study that used physical activity consultation only (and promoted physical activity through walking) among women with postnatal depression was underpowered to detect changes in postnatal depression and like MAMMiS did not demonstrate a significant change in physical activity behaviour at follow-up [48].

The primary outcome for our trial was an objective assessment of moderate to vigorous physical activity (MVPA) [25], and this was not improved by the intervention. However, findings from the qualitative study indicate that many participants perceived an improvement to their physical activity behaviour and related this to perceived improvements in psychological well-being. Studies comparing self-reported versus objectively measured MVPA show that people tend to overestimate the intensity and duration of MVPA, particularly activities performed as part of daily life (e.g., household and caregiving activities) [49]. Thus, the intensity of the additional activity perceived by many participants may have been “light” rather than moderate or vigorous which may explain the discrepancy between the quantitative and qualitative physical activity findings. Alternatively, many participants reported fluctuating physical activity levels (which were seen as an unavoidable consequence of having young children) and felt that accelerometer measurement at the follow-up periods was not fully representative of changes to their physical activity. The psychological gains attributed to being more active were more likely to be proximal outcomes (i.e., concurrent or day-to-day) of importance to the participants. Previous research from a successful physical activity trial has shown that endorsing statements about more immediate outcomes after physical activity (e.g., “feel energized, better overall mood, enjoyment and sense of accomplishment”) [50, page 599] is related to being more active compared to more distal outcome expectancies (e.g., weight loss, fitness change).

While fatigue severity significantly improved in the intervention group compared to the control group (with the control group showing a worsening of fatigue) from baseline to three months, this pattern was reversed between 3 and 6 months. There is some evidence for a positive effect of physical activity on fatigue [9]. This trial recruited women with postnatal depression and improvements were greater among those adhering to the programme [9], highlighting the importance of having an effect on physical activity behaviour.

The participants in the MAMMiS trial were a heterogeneous sample and while randomisation ensured that there were no significant differences between the control and intervention groups it is likely that some of the differences within the groups might affect outcomes. For example, the age of infant, number of siblings, and feeding methods can impact on a mother's ability to take part in physical activity and lose weight. Prepregnancy and current weight and BMI will also affect the potential of a trial to impact on body composition outcomes as suggested by a greater effect in the small number of overweigh/obese women in our trial. Recruiting a more homogenous sample, by either narrowing the inclusion criteria or targeting clinically at-risk groups (e.g., overweight, gestational diabetes, or postnatal depression), might show more benefit from increasing physical activity than in a general healthy postnatal population, although evidence is still inconclusive and to date this has not been tested using objective measures [51, 52]. Furthermore using objective measures for assessing activity levels prior to enrolment might ensure recruitment of a more inactive population. The face-to-face interaction and type of intervention in our trial are likely to appeal to more affluent women who tend to have greater access to social support and fewer environmental and economic barriers to physical activity [53] while E-health or text interventions might reach a more disadvantaged population of postnatal women [54, 55].

### 4.3. Strengths and Limitations

The main strengths of the MAMMiS study were the use of a randomised controlled design, inclusion of an objective measure of physical activity (i.e., accelerometers), and a three-month postintervention follow-up. Prior to the conception and implementation of the trial these methods had not previously been used in physical activity promotion research among postnatal women [24]. The intervention approach used in MAMMiS [27] was theoretically and empirically sound as it had been shown to be effective in other groups and was relevant to research on motivators and barriers to physical change in the postnatal population and the precise content of the intervention was detailed with reference to the behaviour change technique taxonomy in use at the time [56].

The main limitation of the study was that despite attempts to recruit an insufficiently active sample, baseline levels of activity were higher than expected [25]. We used a stage of change questionnaire to screen eligibility prior to baseline measures; this subjective measure is therefore susceptible to self-report bias. It is also likely that our definition of insufficient activity was too high, that is, not achieving five sessions of physical activity per week of at least thirty minutes [30]. In addition, a number of factors can affect estimation of body fat using the bioelectrical impedance method; therefore we controlled for as many of these factors as possible (as described in Section 2); however we were unable to schedule body fat measurements with participants' phase of their menstrual cycle, which may have influenced the body fat results.

## 5. Conclusions and Implications

Although there are substantial health and well-being benefits from participating in regular physical activity during the postnatal period, results from this study (and others) suggest we still lack a definitive approach to increasing physical activity participation among this group.

## Figures and Tables

**Table 1:** Participant baseline sociodemographic and clinical characteristics.

Characteristic^*∗*^	Intervention (*n* = 33)	Control (*n* = 32)
Mean age ± SD, y	33.1 ± 4.1	33.8 ± 5.4

Mean age of youngest child ± SD, weeks (range)	24.0 ± 11.0 (9–48)	24.8 ± 15.5 (7–50)

Median number of children (range)	1 (1–4)	1 (1–5)

Marital status, *n* (%)		
Married/cohabiting	27 (82)/5 (15)	27 (84)/5 (16)
Single	1 (3)	0

Employment status, *n* (%)		
Maternity leave or housewife	31 (94)	24 (74)
Working (full or part time)	2 (6)	5 (16)
Unemployed	0	3 (9)

Breastfeeding status, *n* (%)		
Breast (exclusively or incl. solids)	13 (39)	18 (56)
Bottle (exclusively or incl. solids)	16 (49)	11 (34)
Mixed (can include solids)	4 (12)	3 (9.4)

Method of delivery^*∗∗*^, *n* (%)		
Vaginal labour	24 (73)	26 (81)
Caesarean section	8 (24)	6 (19)

Mean self-reported prepregnancy weight ± SD, kg	65.2 ± 9.9	63.1 ± 8.2

Mean prepregnancy BMI ± SD, kg/m^2^	25.1 ± 4.1	23.6 ± 3.1

Prepregnancy BMI classification, *n* (%)		
Underweight (<18.5 kg/m^2^)	0	1 (3)
Healthy range (18.5–24.9 kg/m^2^)	14 (54)	20 (69)
Overweight (25–29.9 kg/m^2^)	10 (39)	7 (24)
Obese (≥30 kg/m^2^)	2 (8)	1 (3)

Mean measured current weight ± SD, kg	72.9 ± 10.9	68.2 ± 10.4

Mean current BMI ± SD, kg/m^2^	27 ± 4.2	25.5 ± 3.9

Current BMI classification, *n* (%)		
Healthy range (18.5–24.9 kg/m^2^)	13 (39)	18 (56)
Overweight (25–29.9 kg/m^2^)	11 (34)	9 (28)
Obese (≥30 kg/m^2^)	9 (27)	5 (16)

Body mass index, BMI.  ^*∗*^At enrolment. ^*∗∗*^Missing data from one participant from the intervention group.

**Table 2:** Weight and body composition results at baseline and three and six months of follow-up in response to a 10-week physical activity intervention.

	Intervention	Control
	Median (IQ range)	Median (IQ range)
*Weight (kg)*		
Baseline (*n* = 33, 32)	72 (65, 80)	68 (62, 72)
Three months^*∗*^ (*n* = 30)	69 (63, 79)	65 (62, 72)
Six months^*∗∗*^ (*n* = 30,29)	68 (61, 79)	65 (61, 71)

*BMI (kg/m* ^*2*^)		
Baseline (*n* = 33, 32)	27 (24, 30)	25 (22, 27)
Three months^*∗*^ (*n* = 30)	26 (23, 29)	24 (22, 27)
Six months^*∗∗*^ (*n* = 30, 29)	25 (23, 29)	24 (22, 27)

*Fat mass (kg)*		
Baseline (*n* = 33, 31)	26 (20, 33)	22 (18, 26)
Three months^*∗*^ (*n* = 30)	25 (20, 32)	20 (17, 26)
Six months^*∗∗*^ (*n* = 29)	25 (18, 34)	19 (17, 25)

*% fat mass*		
Baseline (*n* = 33, 31)	35 (32, 41)	32 (30, 36)
Three months^*∗*^ (*n* = 30)	35 (35, 40)	31 (29, 35)
Six months^*∗∗*^ (*n* = 29)	34 (29, 41)	30 (27, 35)

BMI, body mass index; IQ range, interquartile range. *n* = numbers in intervention and control group at each measurement time period.

^*∗*^Tested with Mann-Whitney *U* tests not between group differences from baseline to three months for weight (*p* = 0.80), BMI (*p* = 0.80), fat mass (*p* = 0.55), and % fat mass (*p* = 0.81).

^*∗∗*^Tested with Mann-Whitney *U* tests not between group differences three to six months for weight (*p* = 0.84), BMI (*p* = 0.58), fat mass (*p* = 0.66), and % fat mass (*p* = 0.78).

**Table 3:** Psychological well-being at baseline and three and six months in relation to a 10-week physical activity intervention.

Measurement period	Intervention group (*n* = 30)	Control group (*n* = 29)
	Mean (s.d.)	Mean (s.d.)
Baseline	86 (10.6)	90 (8.1)
Three months	89 (9.9)	89 (8.2)
Six months	88 (10.1)	92 (7.5)

Note: the Adapted General Well-Being Index (AGWBI) Likert scale range is 22–110 with higher scores representing more positive well-being.

**Table 4:** Fatigue score at baseline and three and six months in relation to a 10-week physical activity intervention.

Measurement period	Intervention group	Control group
	median (IQ range)	median (IQ range)
Baseline^1^	44 (31, 66)	28 (20, 49)
Three months^2^	26 (15, 58)	49 (26, 61)
Six months^3^	49 (16, 62)	27 (17, 46)

*N* in the intervention (I) and control (C) groups: ^1^I = 33, C = 32, ^2^I = 31, C = 29, ^3^I = 31, and C = 28.

## References

[1] Brown W. J., Pavey T., Bauman A. E. (2015). Comparing population attributable risks for heart disease across the adult lifespan in women. *British Journal of Sports Medicine*.

[2] Gore S. A., Brown D. M., West D. S. (2003). The role of postpartum weight retention in obesity among women: a review of the evidence. *Annals of Behavioral Medicine*.

[3] Hardman A. E., Stensel D. J. (2009). *Physical Activity and Health: The Evidence Explained*.

[4] Weissgerber T. L., Wolfe L. A., Davies G. A. L., Mottola M. F. (2006). Exercise in the prevention and treatment of maternal-fetal disease: a review of the literature. *Applied Physiology, Nutrition and Metabolism*.

[5] Löbner K., Knopff A., Baumgarten A. (2006). Predictors of postpartum diabetes in women with gestational diabetes mellitus. *Diabetes*.

[6] Gilinsky A. S., Kirk A. F., Hughes A. R., Lindsay R. S. (2015). Lifestyle interventions for type 2 diabetes prevention in women with prior gestational diabetes: a systematic review and meta-analysis of behavioural, anthropometric and metabolic outcomes. *Preventive Medicine Reports*.

[7] Dritsa M., Dupuis G., Lowensteyn I., Da Costa D. (2009). Effects of home-based exercise on fatigue in postpartum depressed women: who is more likely to benefit and why?. *Journal of Psychosomatic Research*.

[8] Daley A., Jolly K., MacArthur C. (2009). The effectiveness of exercise in the management of post-natal depression: systematic review and meta-analysis. *Family Practice*.

[9] Sampselle C. M., Seng J., Yeo S., Killion C., Oakley D. (1999). Physical activity and postpartum well-being. *Journal of Obstetric, Gynecologic, and Neonatal Nursing*.

[10] Evenson K. R., Mottola M. F., Owe K. M., Rousham E. K., Brown W. J. (2014). Summary of international guidelines for physical activity after pregnancy. *Obstetrical & Gynecological Survey*.

[11] Albright C. L., Maddock J. E., Nigg C. R. (2006). Physical activity before pregnancy and following childbirth in a multiethnic sample of healthy women in Hawaii. *Women and Health*.

[12] Borodulin K., Evenson K. R., Herring A. H. (2009). Physical activity patterns during pregnancy through postpartum. *BMC Women's Health*.

[13] Grace S. L., Williams A., Stewart D. E., Franche R.-L. (2006). Health-promoting behaviors through pregnancy, maternity leave, and return to work: effects of role spillover and other correlates. *Women and Health*.

[14] Evenson K. R., Herring A. H., Wen F. (2012). Self-reported and objectively measured physical activity among a cohort of postpartum women: the PIN postpartum study. *Journal of Physical Activity and Health*.

[15] Wilkinson S., Huang C.-M., Walker L. O., Sterling B. S., Kim M. (2004). Physical activity in low-income postpartum women. *Journal of Nursing Scholarship*.

[16] Allender S., Hutchinson L., Foster C. (2008). Life-change events and participation in physical activity: a systematic review. *Health Promotion International*.

[17] Bellows-Riecken K. H., Rhodes R. E. (2008). A birth of inactivity? A review of physical activity and parenthood. *Preventive Medicine*.

[18] Brown W. J., Trost S. G. (2003). Life transitions and changing physical activity patterns in young women. *American Journal of Preventive Medicine*.

[19] Vrazel J. E., Saunders R. P., Wilcox S. (2008). An overview and proposed framework of social-environmental influences on the physical-activity behavior of women. *American Journal of Health Promotion*.

[20] Hamilton K., White K. M. (2010). Understanding parental physical activity: meanings, habits, and social role influence. *Psychology of Sport and Exercise*.

[21] Pereira M. A., Rifas-Shiman S. L., Kleinman K. P., Rich-Edwards J. W., Peterson K. E., Gillman M. W. (2007). Predictors of change in physical activity during and after pregnancy: project viva. *American Journal of Preventive Medicine*.

[22] Cramp A. G., Bray S. R. (2009). Pre- and postpartum women's leisure time physical activity patterns: a multilevel longitudinal analysis. *Research Quarterly for Exercise & Sport*.

[23] Evenson K. R., Aytur S. A., Borodulin K. (2009). Physical activity beliefs, barriers, and enablers among postpartum women. *Journal of Women's Health*.

[24] Gilinsky A. S., Dale H., Robinson C., Hughes A. R., McInnes R., Lavallee D. (2015). Efficacy of physical activity interventions in post-natal populations: systematic review, meta-analysis and content coding of behaviour change techniques. *Health Psychology Review*.

[25] Gilinsky A. S. (2014). *Promoting physical activity among postnatal women: the More Active Mums in Stirling (MAMMiS) study [Ph.D. thesis]*.

[26] Guthrie W., Hughes A. R., McInnes R. J., Jepson R., Gilinsky A. S. (2013). The experiences of postnatal women participating in a physical activity intervention: process evaluation of the More Active MuMs in Stirling (MAMMiS) study. *Final Report to the Chief Scientist's Office*.

[27] Gilinsky A. S., Hughes A. R., McInnes R. J. (2012). More Active Mums in Stirling (MAMMiS): a physical activity intervention for postnatal women. Study protocol for a randomized controlled trial. *Trials*.

[29] Yancey A. K., Ortega A. N., Kumanyika S. K. (2006). Effective recruitment and retention of minority research participants. *Annual Review of Public Health*.

[30] Marcus B. H., Rakowski W., Rossi J. S. (1992). Assessing motivational readiness and decision making for exercise. *Health Psychology*.

[31] Haskell W. L., Lee I.-M., Pate R. R. (2007). Physical activity and public health: updated recommendation for adults from the American College of Sports Medicine and the American Heart Association. *Medicine & Science in Sports & Exercise*.

[32] Baker G., Gray S. R., Wright A. (2008). The effect of a pedometer-based community walking intervention ‘Walking for Wellbeing in the West’ on physical activity levels and health outcomes: a 12-week randomized controlled trial. *International Journal of Behavioural Nutrition & Physical Activity*.

[33] Kirk A., Mutrie N., MacIntyre P., Fisher M. (2004). Effects of a 12-month activity counselling intervention on glycaemic control and on the status of cardiovascular risk factors in people with type 2 diabetes. *Diabetologia*.

[34] Hughes A. R., Mutrie N., MacIntyre P. D. (2007). Effect of an exercise consultation on maintenance of physical activity after completion of phase III exercise-based cardiac rehabilitation. *European Journal of Cardiovascular Prevention & Rehabilitation*.

[35] Loughlan C., Mutrie N. (1995). Conducting an exercise consultation: guidelines for health professionals. *International Journal of Health Research*.

[36] (2013). *Tanita 300MA*.

[37] Hunt S. M., McKenna S. P. (1992). A British adaptation of the general well-being index: a new tool for clinical research. *Journal of Medical Economics*.

[38] Hopton J. L., Hunt S. M., Shiels C., Smith C. (1995). Measuring psychological well-being. The adapted general well-being index in a primary care setting: a test of validity. *Family Practice*.

[39] Bowling A. (2005). *Measuring Health: A Review of Quality of Life Measurement Scales*.

[40] Braun V., Clarke V. (2006). Using thematic analysis in psychology. *Qualitative Research in Psychology*.

[41] McElhone S., Kearney J. M., Giachetti I., Zunft H.-J. F., Martínez J. A. (1999). Body image perception in relation to recent weight changes and strategies for weight loss in a nationally representative sample in the European Union. *Public Health Nutrition*.

[42] Deci E. L., Ryan R. M. (2008). Facilitating optimal motivation and psychological well-being across life's domains. *Canadian Psychology*.

[43] Gunderson E. P., Rifas-Shiman S. L., Oken E. (2008). Association of fewer hours of sleep at 6 months postpartum with substantial weight retention at 1 year postpartum. *American Journal of Epidemiology*.

[44] Olson C. M., Strawderman M. S., Hinton P. S., Pearson T. A. (2003). Gestational weight gain and postpartum behaviors associated with weight change from early pregnancy to 1 y postpartum. *International Journal of Obesity*.

[45] Norman E., Sherburn M., Osborne R. H., Galea M. P. (2010). An exercise and education program improves well-being of new mothers: a randomized controlled trial. *Physical Therapy*.

[46] Armstrong K., Edwards H. (2004). The effectiveness of a pram-walking exercise programme in reducing depressive symptomatology for postpartum women. *International Journal of Nursing Practice*.

[47] Armstrong K., Edwards H. (2003). The effects of exercise and social support on mothers reporting depressive symptoms: a pilot randomized controlled trial. *International Journal of Mental Health Nursing*.

[48] Daley A. J., Winter H., Grimmett C., McGuinness M., McManus R., MacArthur C. (2008). Feasibility of an exercise intervention for women with postnatal depression: a pilot randomised controlled trial. *British Journal of General Practice*.

[49] Prince S. A., Adamo K. B., Hamel M. E., Hardt J., Connor Gorber S., Tremblay M. (2008). A comparison of direct versus self-report measures for assessing physical activity in adults: a systematic review. *International Journal of Behavioral Nutrition & Physical Activity*.

[50] Cramp A. G., Brawley L. R. (2009). Sustaining self-regulatory efficacy and psychological outcome expectations for postnatal exercise: effects of a group-mediated cognitive behavioural intervention. *British Journal of Health Psychology*.

[51] Daley A. J., Jolly K., Sharp D. J. (2012). The effectiveness of exercise as a treatment for postnatal depression: study protocol. *BMC Pregnancy and Childbirth*.

[52] Bennett W. L., Liu S.-H., Yeh H.-C. (2013). Changes in weight and health behaviors after pregnancies complicated by gestational diabetes mellitus: the CARDIA study. *Obesity*.

[53] Kim C., McEwen L. N., Kieffer E. C., Herman W. H., Piette J. D. (2008). Self-efficacy, social support, and associations with physical activity and body mass index among women with histories of gestational diabetes mellitus. *The Diabetes Educator*.

[54] Huberty J., Rowedder L., Hekler E. (2016). Development and design of an intervention to improve physical activity in pregnant women using Text4baby. *Translational Behavioral Medicine*.

[55] Gray S. (2014). Move more mommy: a web-based physical activity intervention for postnatal women (pilot study). *Electronic Thesis and Dissertation Repository*.

[56] Michie S., Ashford S., Sniehotta F. F., Dombrowski S. U., Bishop A., French D. P. (2011). A refined taxonomy of behaviour change techniques to help people change their physical activity and healthy eating behaviours: the CALO-RE taxonomy. *Psychology and Health*.

